# Comparative Impact of Coronary Imaging Strategies in CTO-PCI: A Retrospective Single-Center Analysis

**DOI:** 10.3390/jcm14196976

**Published:** 2025-10-01

**Authors:** Giuseppe Panuccio, Kambis Mashayekhi, Gerald S. Werner, Yasuhiro Ichibori, Nicole Carabetta, Carsten Skurk, Ömer Göktekin, Patrick T. Siegrist, David M. Leistner, Salvatore De Rosa, Daniele Torella, Ulf Landmesser, Youssef S. Abdelwahed

**Affiliations:** 1 Department of Experimental and Clinical Medicine, Magna Graecia University, 88100 Catanzaro, Italy; 2 Department of Cardiology, Angiology and Intensive Care Medicine, Deutsches Herzzentrum der Charite, 12203 Berlin, Germany; 3 Internal Medicine and Cardiology, Heart Center Lahr, 77933 Lahr, Germany; 4 Department of Cardiology, Heart Center, University Hospital, Goethe University Frankfurt, 60629 Frankfurt, Germany; 5 Osaka Police Hospital, Osaka 543-0042, Japan; 6 Department of Medical and Surgical Sciences, Magna Grecia University, 88100 Catanzaro, Italy; 7 DZHK (German Centre for Cardiovascular Research), 10785 Berlin, Germany; 8 Memorial Bahcelievler Hospital, Istanbul 34180, Turkey; ogoktekin@gmail.com; 9 HerzZentrum Hirslanden Zurich, CH-8008 Zürich, Switzerland; 10 DZHK Partner Site Rhine-Main, 60590 Frankfurt, Germany; 11 Cardio-Pulmonary Institute, Partner Site Frankfurt, 60590 Frankfurt, Germany; 12 Berlin Institute of Health (BIH), 10178 Berlin, Germany

**Keywords:** cardiac imaging, chronic total occlusions, cardiac computed tomography angiography, intravascular ultrasound

## Abstract

**Background:** Coronary imaging is increasingly used in chronic total occlusion percutaneous coronary intervention (CTO-PCI), but the impact of different imaging strategies on procedural decisions and outcomes remains unclear. **Methods:** We retrospectively analyzed 171 consecutive patients undergoing CTO-PCI, stratified by imaging strategy into four groups: angiography-only (n = 48), IVUS-guided (n = 42), CT-guided (n = 40) and CT + IVUS-guided (n = 41). Procedural and in-hospital clinical outcomes were compared. A multivariable logistic regression identified predictors of intense debulking techniques (defined as the use of rotational atherectomy or intravascular lithotripsy). **Results:** Imaging guidance was associated with progressively longer procedural (*p* < 0.001) and fluoroscopic time (*p* = 0.007). Similarly, an increased number of guidewires (*p* = 0.005) and balloons (*p* = 0.003) was used in the imaging groups, with the CT + IVUS groups showing the highest features. Regarding stenting characteristics, higher stent length and diameter (*p* = 0.01) were observed in the imaging groups. In patients with J-CTO score > 2, procedural success rates significantly increased with the use of coronary imaging (*p* = 0.01). Multivariable analysis showed that both J-CTO score (OR 2.0; 95% CI 1.3–3.0; *p* = 0.001) and imaging strategies (OR 1.6; 95% CI 1.02–2.4; *p* = 0.04) independently predicted the use of intense debulking techniques. Importantly, no significant differences were observed in in-hospital complications across groups. **Conclusions:** The use of coronary imaging, particularly the combination of IVUS and CT, is associated with more complex CTO lesions and led to increased procedural time, fluoroscopic time and more extensive stenting, as well as higher debulking usage. In complex CTO cases, coronary imaging was associated with higher procedural success rates. Imaging strategies independently predicted the need for advanced lesion preparation, beyond anatomical complexity, without compromising safety. Despite higher procedural demands, coronary imaging enables a more tailored and successful approach to CTO-PCI, particularly in complex cases. These findings underscore the pivotal role of multimodal imaging in the procedural planning and optimization of CTO-PCI.

## 1. Introduction

Chronic total occlusions (CTOs) represent one of the most challenging subsets in percutaneous coronary interventions (PCI) [1,2,3,4,5,6,7]. CTO-PCI procedures are often associated with longer procedural times and radiation exposure, and with usage of different approaches from conventional PCI—such as extra-plaque or retrograde techniques as well as operator expertise [8,9,10,11]. In this setting, intracoronary imaging, particularly intravascular ultrasound (IVUS), has emerged as a valuable tool to improve procedural planning, optimize stent implantation and improve outcomes [12,13,14,15]. In particular, several studies have shown a reduction in major adverse cardiac events (MACE) compared to angiography alone in complex lesions, including CTOs [16]. More recently, the integration of coronary computed tomography angiography (CCTA) into pre-procedural planning has shown promise in characterizing CTO morphology, vessel course and proximal cap ambiguity in more detail, potentially improving crossing strategies and procedural outcomes [17,18]. However, despite the increasing use of both IVUS and CCTA in CTO interventions, current evidence on the impact of different imaging strategies—alone or in combination—is limited. No studies to date have directly compared angiography-guided CTO-PCI with IVUS-guided and combined IVUS + CCTA-guided strategies in a real-world setting. Therefore, the aim of this study is to address this knowledge gap comparing the procedural and in-hospital outcomes of three different imaging approaches during CTO-PCI.

## 2. Methods

This was a retrospective, single-center observational study conducted at a high-volume European tertiary center. We included 171 patients who underwent elective CTO-PCI at the Deutsches Herzzentrum der Charitè, Berlin, Germany, between 2019 and 2024. Patients were divided into four groups according to the imaging strategy employed during CTO-PCI procedure: (1) angiography-guided only; (2) IVUS-guided; (3) CCTA-guided; (4) combined IVUS + CCTA-guided. The choice of imaging strategy was at the discretion of the operating physician and based on clinical, anatomical and procedural considerations. The inclusion criteria were age ≥ 18 years, and elective CTO-PCI for at least one native coronary artery CTO (defined as TIMI 0 flow for >3 months or unknown duration). CTO-PCI procedures were performed according to the current European Society of Cardiology (ESC) guidelines [19]. Exclusion criteria were myocardial infarction (MI) within 48 h, cardiogenic shock, CTOs in bypass grafts and suboptimal quality of CCTA images.

### 2.1. Imaging Guidance Definitions

Angiography-guided was defined as procedural planning based solely on fluoroscopic angiographic images. IVUS-guided was defined as use of intravascular ultrasound during PCI, including assessment of lesion morphology, stent sizing and optimization. CCTA + IVUS guidance was characterized by pre-procedural CCTA (within six weeks before CTO-PCI) used for procedural planning (vessel course, CTO characteristics, cap identification), combined with intra-procedural IVUS guidance. IVUS was employed for intimal tracking, pre-stent lesion assessment and post-stent optimization. Pullback analysis was performed when technically feasible. Optimization criteria included assessment of minimum stent area (MSA), stent expansion, apposition and the presence of edge dissections. Optimization criteria included achieving a minimum stent area (MSA) ≥ 5.5 mm^2^ or ≥90% of the distal reference lumen area, absence of major malapposition and no major edge dissection [20].

CCTA images were analyzed using a specialized workstation (Synapse 3D, Fujifilm). The thin slab maximum intensity projection (MIP) and curved and stretched multiplanar reconstruction (MPR) were used to evaluate the anatomy of the CTO vessel. CCTA images were used for preprocedural planning, specifically for identifying proximal cap ambiguity, vessel course, tortuosity, calcification patterns and evaluation of distal landing zones. The selection for each imaging group was at the operator’s discretion. In particular, the use of CCTA was often based according to complexity features such as prior failed attempts and poor distal visualization on angiography.

### 2.2. Study Endpoints

Experienced CTO operators with more than 300 CTO-PCI cases as first operator performed all PCI procedures following European CTO Club strategies and standards [21]. The primary endpoints were procedural time (from first vascular access to last angiographic image), fluoroscopic time and contrast volume. Secondary endpoints were the number of guidewires and balloons used, stent number, length and diameter, use of advanced lesion preparation techniques (intravascular lithotripsy or rotational atherectomy), in-hospital MACE (defined as in-hospital death, myocardial infarction or clinically driven target vessel revascularization [22], coronary perforations and post-procedural creatinine levels. Contrast-associated acute kidney injury (CA-AKI) was defined as an absolute increase in serum creatinine ≥ 0.5 mg/dL or a relative increase ≥ 25% within 48–72 h after the procedure. Data were extracted from the institutional CTO-PCI registry and cross-verified with procedural and imaging reports.

### 2.3. Statistical Analysis

Continuous variables are presented as mean ± standard deviation or median with interquartile range, as appropriate. We used Q-Q (quantile-quantile) plots and the Shapiro–Wilk test to determine the distribution of the continuous data. Consequently, the Student’s t-test or the Mann–Whitney U test was used, as appropriate, to compare continuous data. Categorical variables were reported as counts and percentages. Group comparisons were performed using one-way ANOVA or Kruskal–Wallis test for continuous variables and chi-square or Fisher’s exact test for categorical variables. Post-hoc pairwise comparisons were performed using Bonferroni correction for parametric variables and Dunn’s test with Bonferroni adjustment for non-parametric variables. Procedural outcomes were further analyzed using analysis of covariance (ANCOVA), and covariate balance was assessed using standardized mean differences (SMDs) before and after inverse probability of treatment weighting (IPTW). Multivariate logistic regression was used to identify independent predictors of the use of advanced debulking techniques. The effect size was quantified using Odds Ratios (OR) with 95% CI. All the tests are two-sided, and a *p* value < 0.05 was considered statistically significant. Analyses were performed using the Statistical Package for the Social Sciences (SPSS), version 25 (IBM Corp., Armonk, NY, USA).

## 3. Results

**Figure 1:**
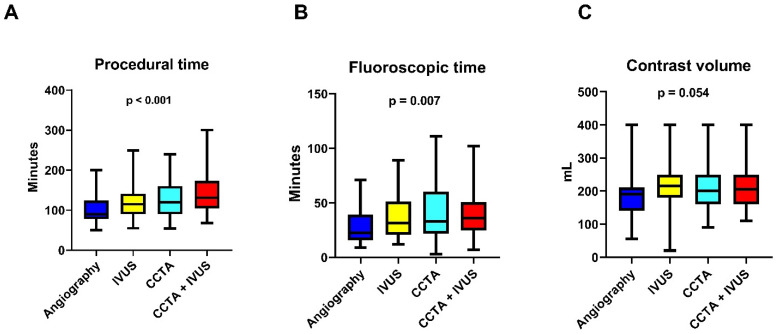
Comparison of procedural time, fluoroscopic time and contrast volume across the four study groups: angiography-guided, IVUS-guided, CCTA-guided and combined CCTA + IVUS-guided. Data are shown as median and interquartile range. *p* values are from Kruskal–Wallis test; post-hoc pairwise comparisons were adjusted using Dunn’s test with Bonferroni correction. Post-hoc comparisons: (**A**) procedural time: angiography vs. IVUS *p* = 0.07; angiography vs. CCTA *p* = 0.02; angiography vs. CCTA + IVUS *p* < 0.001; IVUS vs. CCTA *p* = 0.99; IVUS vs. CCTA + IVUS *p* = 0.47; CCTA vs. CCTA + IVUS *p* = 0.99; (**B**) fluoroscopic time: angiography vs. IVUS *p* = 0.10; angiography vs. CCTA *p* = 0.04; angiography vs. CCTA + IVUS *p* = 0.01; IVUS vs. CCTA *p* = 0.99; IVUS vs. CCTA + IVUS *p* = 0.99; CCTA vs. CCTA + IVUS *p* = 0.99; (**C**) contrast volume: angiography vs. IVUS *p* = 0.04; angiography vs. CCTA *p* = 0.61; angiography vs. CCTA + IVUS *p* = 0.34; IVUS vs. CCTA *p* = 0.99; IVUS vs. CCTA + IVUS *p* = 0.99; CCTA vs. CCTA + IVUS *p* = 0.99. All the findings remained consistent after multivariable adjustment for age and J-CTO score.

**Figure 2:**
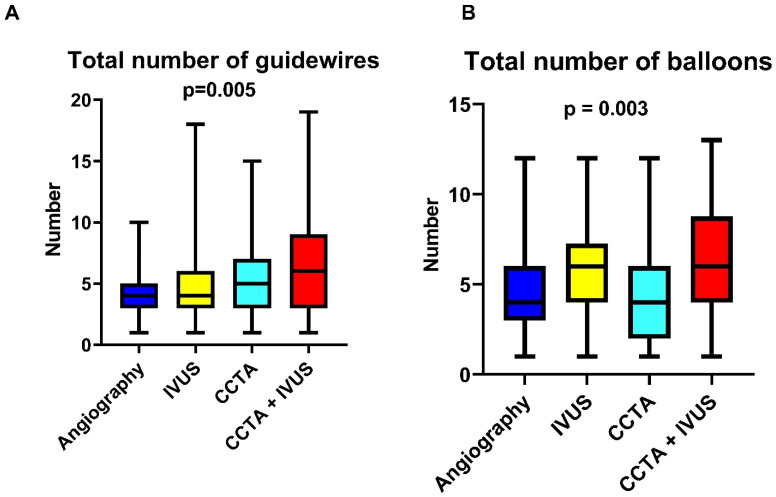
Comparison of number of guidewires and balloons used across the four groups. Data are shown as median and interquartile range. *p* values are from Kruskal–Wallis test; post-hoc pairwise comparisons were adjusted using Dunn’s test with Bonferroni correction. Post-hoc comparisons: (**A**) number of guidewires: angiography vs. IVUS *p* = 0.99; angiography vs. CCTA *p* = 0.04; angiography vs. CCTA + IVUS *p* = 0.008; IVUS vs. CCTA *p* = 0.83; IVUS vs. CCTA + IVUS *p* = 0.30; CCTA vs. CCTA + IVUS *p* = 0.99; (**B**) number of balloons: angiography vs. IVUS *p* = 0.08; angiography vs. CCTA *p* = 0.99; angiography vs. CCTA + IVUS *p* = 0.01; IVUS vs. CCTA *p* = 0.18; IVUS vs. CCTA + IVUS *p* = 0.99; CCTA vs. CCTA + IVUS *p* = 0.04. All the findings remained consistent after multivariable adjustment for age and J-CTO score.

**Figure 3:**
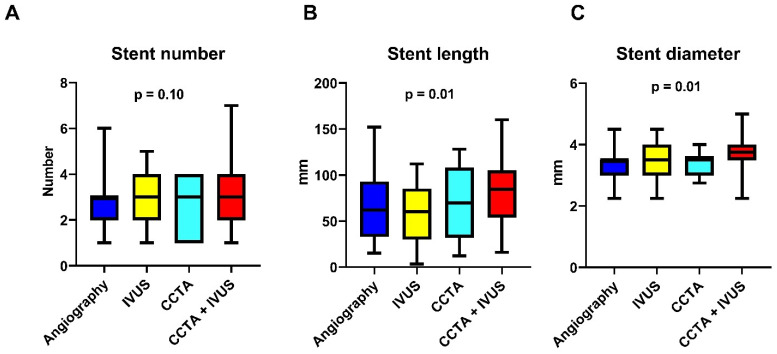
Comparison of stent number, length and diameter across the four groups. Data are shown as median and interquartile range. *p* values are from Kruskal–Wallis test; post-hoc pairwise comparisons were adjusted using Dunn’s test with Bonferroni correction. Post-hoc comparisons: (**A**) stent number: angiography vs. IVUS *p* = 0.99; angiography vs. CCTA *p* = 0.99; angiography vs. CCTA + IVUS *p* = 0.10; IVUS vs. CCTA *p* = 0.99; IVUS vs. CCTA + IVUS *p* = 0.98; CCTA vs. CCTA + IVUS *p* = 0.64; (**B**) stent length: angiography vs. IVUS *p* = 0.99; angiography vs. CCTA *p* = 0.99; angiography vs. CCTA + IVUS *p* = 0.18; IVUS vs. CCTA *p* = 0.99; IVUS vs. CCTA + IVUS *p* = 0.01; CCTA vs. CCTA + IVUS *p* = 0.99; (**C**) stent diameter: angiography vs. IVUS *p* = 0.99; angiography vs. CCTA *p* = 0.99; angiography vs. CCTA + IVUS *p* = 0.01; IVUS vs. CCTA *p* = 0.99; IVUS vs. CCTA + IVUS *p* = 0.12; CCTA vs. CCTA + IVUS *p* = 0.07. All the findings remained consistent after multivariable adjustment for age and J-CTO score.

**Figure 4:**
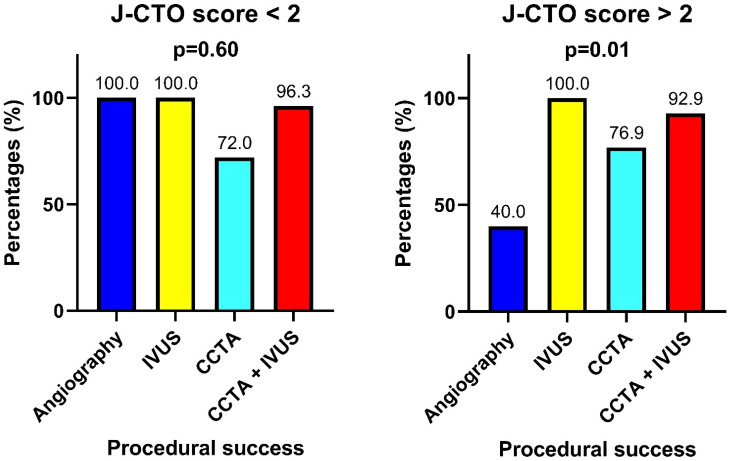
Subgroup analysis of procedural success between imaging strategies according to J-CTO score.

A total of 171 patients undergoing elective CTO-PCI were included in the analysis. Patients were stratified into four groups based on the imaging guidance strategy: angiography-based only (n = 48), IVUS-guided (n = 42), CCTA-guided (n = 40) and combined CCTA + IVUS-guided (n = 41). Baseline characteristics were comparable between groups, although patients in the imaging-guided arms had more complex lesions, including higher J-CTO and EURO-CTO (CASTLE) scores (Table 1). The main reasons for the use of coronary imaging were proximal cap ambiguity (10 patients, 5.8% of the total), guidance in sub-intimal tracking (10 patients, 66% of the patients treated with antegrade dissection and re-entry), evaluation of calcium distribution (123 patients, 71.9%) and intense debulking usage (22 patients, 91.6% of patients treated with intense debulking techniques). Procedural success was achieved in 154 patients (90.0%). The main reasons for procedural failure were the inability to cross the lesion with guidewires or microcatheters (n = 15; 88.2%), perforation (n = 1; 5.8%) and rotational atherectomy burr entrapment (n = 1; 5.8%). Procedural time was significantly longer in both the IVUS-guided, CCTA-guided and CCTA + IVUS-guided groups compared to the angiography-only group (90 [78.25–123.75] vs. 115 [90–141.25] vs. 120 [90–160] vs. 131 [105–173]; p < 0.001; Figure 1A). Post-hoc comparisons confirmed a significant difference between angiography and IVUS (*p* = 0.02), angiography and CCTA (*p* = 0.03) and between angiography and CCTA + IVUS (*p* < 0.001). In contrast, no significant difference was observed between the imaging-guided groups. Similarly, fluoroscopy time was higher in the imaging-guided groups (22.5 [16–39] vs. 31.5 [20.75–51] vs. 33 [22–60] vs. 36 [25–50.5]; *p* = 0.007; Figure 1B). Post-hoc analysis showed a significant increase in fluoroscopic time for both CCTA (*p* = 0.04) and CCTA + IVUS group (*p* = 0.003) compared to angiography, with no significant differences between imaging-guided groups. Contrast volume tended to be higher in the IVUS group (190 [141–210] in the angiography-guided group vs. 215 [180–249.25] in the IVUS group vs. 300 [160–249] in the CCTA group vs. 205 [160–249] in the CCTA + IVUS group; *p* = 0.054; Figure 1C). Post-hoc pairwise comparisons suggested a nominal difference between angiography and IVUS (*p* = 0.04), although the global test did not reach statistical significance. The number of guidewires [30–85 used increased across the groups (4 [3–5] in the angiography group, 4 [3–6] in the IVUS group, 5 [3–7] in the CCTA group and 6 [3–9] in the CCTA + IVUS group; *p* = 0.005; Figure 2A) as did the number of balloons (4 [3–6] in the angiography group; 6 [4–7.25] in the IVUS group, 4 [2–6] in the CCTA group and 6 [4–8.25] in the CCTA + IVUS group; *p* = 0.003; Figure 2B). Regarding stent strategy, stent length was higher in the imaging-guided groups (60 [29.5–91.3] in the angiography group vs. 69 [30–85] in the IVUS group vs. 69.5 [32–108] in the CCTA group vs. 84.5 [54–105.5] in the CCTA + IVUS group; *p* = 0.01; Figure 3B), and diameter (3.5 [3–3.5] in the angiography group vs. 3.5 [3,4] in the IVUS group vs. 3.5 [3–3.625] in the CCTA group vs. 3.75 [3.5–4] in the CCTA + IVUS group; *p* = 0.01; Figure 3C). Stent length was significantly higher in the CCTA + IVUS group with respect to the IVUS group (*p* = 0.01), whereas no significant differences were observed with the other groups. Finally, stent diameter was significantly higher in the CCTA + IVUS group with respect to the angiography group (*p* = 0.01), and there were no significant differences with the other groups. All findings remained consistent after adjustment for age and J-CTO scores in multivariable analysis. Before adjustment, age and J-CTO were unbalanced between groups (SMDs up to 0.58). After weighting, SMDs for both covariates were <0.10, indicating good balance. Adjusted analyses confirmed statistical significance for procedural time (*p* = 0.002), fluoroscopic time (*p* = 0.04), total number of guidewires (*p* = 0.01) and balloons (*p* = 0.01). The adjusted analyses also confirmed the differences in stent length (*p* = 0.02) and diameter (*p* = 0.01). Accordingly, no significant differences were observed across groups for contrast volume (*p* = 0.14) and stent number (*p* = 0.49). Subgroup analysis according to J-CTO score showed that in patients with J-CTO score > 2 the use of coronary imaging was associated with significantly higher rates of procedural success in respect to angiography-only guidance (40% vs. 100% vs. 76.9% vs. 92.9%; *p* = 0.01; Figure 4). Detailed procedural outcomes data are provided in Table 2. In multivariable logistic regression analysis, both J-CTO score (OR 2.0; 95% CI 1.3–3.0; *p* = 0.001) and coronary imaging strategies (OR 1.6; 95% CI 1.02–2.4; *p* = 0.04) independently predicted the use of intense debulking techniques (including rotational atherectomy and intravascular lithotripsy, Table 3). The main reasons for the use of intense debulking were balloon uncrossable lesions or poor balloon expansion. Debulking device-related complications included coronary perforations (n = 1) and rotational atherectomy burr entrapment (n = 1). No significant differences were found in in-hospital MACE among the four groups (2.0% in the angiography-guided group vs. 2.4% in the CCTA + IVUS-guided group; *p* = 0.9). Similarly, the incidence of coronary perforation was comparable among groups (2.0% in the angiography-guided group, vs. 4.7% in the IVUS group vs. 12.5% in the CCTA group and 4.8% in the CCTA + IVUS group; *p* = 0.42). Finally, post-procedural serum creatinine levels were also similar (1.1 [0.97–1.64] vs. 1.16 [0.91–1.36] vs. 1.3 [1.1–1.6] vs. 1.06 [0.89–1.5]; *p* = 0.77), with no evidence of contrast-induced nephropathy (Table 4).

## 4. Discussion

In this study, we investigated the safety and the impact of four different imaging strategies—angiography alone, IVUS-guided, CCTA-guided and the combination of CCTA guidance and IVUS on procedural and in-hospital outcomes in patients undergoing CTO-PCI. Our findings show that the increasing use of coronary imaging was linked to greater procedural complexity and more extensive device use, including debulking techniques, but without compromising safety. The use of coronary imaging guidance approaches was associated with significantly and progressively higher procedural time, fluoroscopic time, contrast volume and number of guidewires and balloons. These findings reflect not only the additional time required to perform and interpret imaging, but also the influence of imaging on operators’ behavior. Interpretation of CCTA images was performed before the procedure, therefore without influencing procedural time. However, CCTA + IVUS-guided groups showed consistently higher procedural features, confirming the impact of coronary imaging in guiding operators’ behavior. Stent strategies were also affected: stent number, length and diameter were significantly higher in the imaging-guided groups, with the CT + IVUS group showing the highest features. Indeed, post-hoc pairwise comparison confirmed that most differences were driven by the CCTA + IVUS group, suggesting a stepwise escalation of procedural complexity in parallel with multimodal imaging usage. Notably, procedural success was not analyzed as a primary endpoint, since imaging guidance—particularly IVUS—is typically employed after successful CTO crossing. Therefore, comparing success rates would be inherently biased and not methodologically appropriate. One of the main findings of our study is that both J-CTO score and coronary imaging strategy were independent predictors of advanced debulking techniques (rotational atherectomy or intravascular lithotripsy). These findings underline that coronary imaging not only provides insights into CTO complexity but also actively influences procedural decision-making in CTO-PCI [23]. IVUS contributes primarily by providing real-time intraprocedural information regarding plaque composition, vessel size and calcium distribution, which supports accurate stent sizing and optimization [24,25]. In contrast, preprocedural CCTA plays a key role in the morphological and spatial characterization of the CTO lesion, allowing precise visualization of proximal cap ambiguity, vessel course, length of calcification and especially diffuse calcification and vessel tortuosity, which may not be fully appreciated on angiography [26,27]. This preprocedural insight can prompt operators to adopt a more tailored and intense lesion preparation strategy. To our knowledge, this is the first study to show a quantitative association between a graded imaging strategy and the use of debulking in CTO-PCI. Although the combination of multimodality imaging was associated with longer procedural times and increased use of materials, our findings suggest that this additional resource utilization is clinically justified. While our findings may rise concerns regarding cost and time, such increase was paralleled by higher procedural success rates, particularly in more complex cases, and a similar rate of in-hospital complications. These findings suggest that the added resources may be justified in selected patients, where tailored imaging guidance enables a more effective revascularization. The significant increase in procedural success observed in complex CTO lesions (J-CTO > 2) further supports the rationale for using imaging-guided approaches in challenging cases. However, given the retrospective, non-randomized design of our study, our findings represent associations rather than proof of causality. The higher procedural success observed in the J-CTO > 2 subgroup, should be interpreted with caution, as it may partly reflect selection bias. Despite increased procedural complexity in the imaging groups, in-hospital outcomes were similar across all strategies. Although myocardial injury markers were not systematically available in our cohort, cardiac biomarkers remain important indicators of periprocedural myocardial injury and their systematic assessment could provide additional insights, as highlighted in previous studies [28]. No significant differences were observed in MACE, coronary perforation or post-procedural creatinine serum levels. These data support the procedural safety of both IVUS and CCTA-guided CTO-PCI, even in anatomically challenging cases [29]. This is consistent with prior randomized trials, such as IVUS-XPL and CTO-IVUS, which showed favorable safety profiles for IVUS-guided PCI in complex lesions [30,31]. Since randomized trials and prospective registries have already demonstrated the individual benefits of IVUS and CCTA, our aim was to provide exploratory evidence on the impact of their combined use in CTO-PCI. While the benefit of IVUS in optimizing PCI outcomes has been widely demonstrated, especially in long or complex lesions, the role of CCTA in procedural decision-making is less well-defined. Previous studies have focused on the role of CCTA in crossing strategy planning, proximal cap identification and vessel course visualization [32]. Our study extends this knowledge by demonstrating that the integration of CCTA into the procedural workflow—when combined with IVUS—has a significant effect on changes in procedural behavior and equipment selection. These findings expand on recent literature and support the adoption of multimodality imaging in selected CTO cases.

## 5. Limitations

This study has some limitations. First, it is a single-center retrospective and non-randomized analysis, which may be affected by selection and information bias. Baseline characteristics were not balanced across groups, as patients undergoing coronary imaging generally had more complex lesions. This imbalance may have influenced the association between imaging and outcomes, despite adjustment for factors such as age and J-CTO scores showed consistent results. Furthermore, the sample size of this study is relatively small, and this may have influenced the generalizability of our findings. However, at the same time, this reflects the highly selected population of CTO-PCI patients undergoing multimodality imaging, warranting future investigation for its application. Second, the imaging strategy was not randomized but left to the operator’s discretion, potentially introducing confounding. In addition, the relatively small number of patients in the related subgroups further limits the generalizability of our results. Moreover, the findings regarding procedural time should be contextualized in the CTO-PCI field and interpreted with caution, as this endpoint is influenced by several factors including lesion complexity, operators’ technique and the use of coronary imaging. Despite fluoroscopic time is a standardized procedural metric in CTO-PCI, other important features such as Air Kerma (AK) and Dose-Area-Product (DAP) were not available for the present analysis. Additionally, the subgroup analysis stratified by lesion complexity (J-CTO score) was likely underpowered due to the limited number of patients within each stratum. Third, although multivariable adjustment was applied, the number of events of intense debulking usage was relatively small, limiting the number of covariates in the model. Additionally, rotational atherectomy and intravascular lithotripsy were grouped together in the intense debulking endpoint, which could be a simplification that limits the applicability of the related findings. Fourth, the study focused on procedural and in-hospital outcomes only; outcomes like procedural success were reported only descriptively and long-term clinical outcomes like MACE remain to be assessed in prospective studies. However, we are currently collecting long-term follow up data on MACE, cardiovascular mortality, target lesion and target vessel revascularization, to assess whether the procedural features observed with coronary imaging strategies translate into durable clinical benefit over time. Therefore, our results should be primarily interpreted as hypothesis-generating, and larger studies with robust propensity methods or randomization are needed to confirm these findings.

## 6. Conclusions

Multimodality imaging in CTO-PCI, particularly the combination of IVUS and CCTA, was associated with increased procedural complexity and more intensive lesion preparation. Despite longer procedural time and higher device usage, imaging-guided strategies led to higher procedural success in complex CTO lesions. They proved to be safe, with no increase in in-hospital adverse events. These findings highlight the clinical value of multimodality imaging, providing anatomical and procedural insights that support more tailored and optimized treatment strategies. Further prospective studies are warranted to evaluate the long-term benefits of imaging-guided approaches in CTO interventions.

## Figures and Tables

**Table 1:** Baseline characteristics of the study patients. IVUS: intravascular ultrasound; CCTA: coronary computed tomography angiography.

Baseline Characteristics	All N = 171	Angiography-Guided N = 48	IVUS-Guided N = 42	CCTA-Guided N = 40	CT+ IVUS-Guided N = 41	*p* Value
Age	68 [60–76]	70.5 [61.2–78]	71.5 [63.7–79]	67 (59–74)	67.0 [59.5–73]	0.13
Male sex	130 (76)	33 (68.7)	33 (78.5)	33 (82.5)	31 (75.6)	0.48
Hypertension	118 (69))	33 (68.7)	29 (69.0)	27 (67.5)	29 (70.7)	0.54
Dyslipidemia	122 (71.3)	35 (72.9)	31 (73.8)	26 (65)	30 (73.1)	0.63
Diabetes	60 (35)	11 (22.9)	19 (45.2)	16 (40)	14 (34.1)	0.16
Smoking	48 (28)	13 (27.0)	8 (19.0)	14 (35)	13 (31.7)	0.35
Peripheral artery disease (PAD)	14 (8)	1 (2.0)	4 (9.5)	5 (12.5)	4 (9.7)	0.24
COPD	12 (7.0)	3 (6.2)	3 (7.1)	5 (12.5)	1 (2.4)	0.59
Chronic kidney disease (CKD)	22 (12.9)	6 (12.5)	6 (14.2)	6 (15)	4 (9.7)	0.52
Prior stroke	7 (4)	1 (2.0)	3 (7.1)	2 (5)	1 (2.4)	0.39
Previous MI	59 (34.5)	19 (39.5)	16 (38.0)	12 (30)	12 (29.2)	0.50
Previous PCI	66 (38.5)	16 (33.3)	22 (52.3)	14 (35)	14 (34.1)	0.08
LVEF	51.50 [40–60]	55 [43.75–60]	53 [36.25–63]	52 (43–60)	47 [40–55]	0.56
Prior CABG	38 (22.2)	4 (8.3)	6 (14.2)	14 (35)	14 (34.1)	**0.009**
Syntax score	24 [18–31]	20 [15–26]	23 [18–29]	27 [21–33]	29 [23–26]	**0.006**
**CTO Artery**						
Right coronary artery	92 (53.8)	25 (52.0)	17 (40.4)	22 (55)	28 (68.2)	0.18
Left anterior descending artery	39 (22.8)	10 (20.8)	15 (35.7)	8 (20)	6 (14.6)	0.89
Left circumflex	37 (21.6)	12 (25.0)	8 (19.0)	10 (25)	7 (17.0)	0.13
CTO location						
Ostial	23 (13.4)	7 (14.5)	6 (14.2)	7 (17.5)	3 (7.3)	0.05
Proximal	91 (53.21)	28 (58.3)	16 (38.0)	21 (52.5)	26 (63.4)	0.16
Mid	45 (26.3)	10 (20.8)	16 (38.1)	10 (25)	9 (22)	0.23
Distal	12 (7)	3 (6.2)	4 (9.5)	2 (5)	3 (7.3)	0.45
In-stent CTO	35 (20.4)	7 (14.5)	14 (33.3)	9 (22.5)	5 (12.1)	0.06
Bifurcation involvement	38 (22.2)	7 (14.5)	13 (30.9)	12 (30)	6 (14.6)	0.56
Stump morphology	66 (38.5)	15 (31.2)	15 (35.7)	14 (35)	22 (53.6)	0.08
Calcification	99 (57.8)	24 (50.0)	24 (57.1)	24 (60)	27 (65.8)	0.13
Previous attempts	13 (7.6)	1(2)	3 (7)	4 (10)	5 (12.1)	0.56
Radial access	161 (94.1)	46 (95.8)	39 (92.8)	37 (92.5)	39 (95.1)	0.25
Contralateral injection	50 (29.2)	11 (22.9)	10 (23.8)	13 (32.5)	16 (39.0)	0.29
Dual antiplatelet therapy	171 (100)	48 (100)	42 (100)	40 (100)	41 (100)	1.0
IVUS	83 (48.53)	0	42 (100)	0	41 (100)	0.42
Antegrade recanalization	166 (97)	46 (95.8)	42 (100)	38 (95)	40 (97.6)	0.40
Antegrade dissection and re-entry	17 (9.9)	5 (10.4)	1 (2.3)	2 (5)	9 (21.9)	**0.01**
Parallel wire	10 (5.8)	3 (6.2)	1 (2.3)	5 (12.5)	1 (2.4)	0.15
Retrograde recanalization	6 (3.5)	0	1 (2.3)	3 (7.5)	2 (4.9)	0.30
J-CTO score	1.7 ± 1.1	1.38 ± 0.9	1.64 ± 1.1	1.98 ± 1.1	1.95 ± 1.3	**0.03**
EURO-CTO score	2.2 ± 1.2	1.95 ± 1.0	2.12 ± 1.3	2.33 ± 1.1	2.31 ± 1.2	0.65
KCCT score	3.05 ± 1.2			2.93 ± 1.1	3.20 ± 1.4	0.35
CT-RECTOR score	1.87 ± 0.8			1.70 ± 0.7	2.06 ± 0.9	0.06

**Table 2:** Procedural outcomes comparisons between different strategies. IVUS: intravascular ultrasound; CCTA: coronary computed tomography angiography.

Outcome	All N = 171	Angiography-Guided	IVUS-Guided	CCTA-Guided	CCTA + IVUS-Guided	*p* Value
Procedural time	115 [90–150]	90 [78.25–123.75]	115 [90–141.25]	120 [90–160]	131 [105–173]	**<0.001**
Fluoroscopic time	30 [20.0–44.0]	22.5 [16–39]	31.5 [20.75–51]	33 [22–60]	36 [25–50.5]	**0.007**
Contrast volume	200 [169–244.25]	190 [141–210]	215 [180–249.25]	200 [160–249]	205 [160–249]	0.054
Stent number	3 [2–4]	3 [2–3]	3 [1.75–4]	3 [1–4]	3 [2–4]	0.10
Stent length	70.5 [38.25–98.0]	60 [32–91.5]	60 [30–85]	69.5 [32–108]	84.5 [54–104.75]	**0.01**
Stent diameter	3.5 [3–4]	3.5 [3–3.5]	3.5 [3–4]	3.5 [3–3.625]	3.75 [3.5–4]	**0.01**
Total number of guidewires	5 [3–7]	4 [3–5]	4 [3–6]	5 [3–7]	6 [3–9]	**0.005**
Total number of balloons	5 [3–7]	4 [3–6]	6 [4–7.25]	4 [2–6]	6 [4–8.75]	**0.003**

**Table 3:** In-hospital outcomes comparison between different strategies. IVUS: intravascular ultrasound; CCTA: computed tomography angiography.

Outcome	All N = 171	Angiography-Guided N = 48	IVUS-Guided N = 42	CCTA-Guided N = 40	CT + IVUS-Guided N = 41	*p* Value
Procedural success	154 (90.0)	45 (93.8)	42 (100)	28 (70.0)	39 (95.1)	0.97
Coronary perforations	10 (5.8)	1 (2.0)	2 (4.7)	5 (12.5)	2 (4.8)	0.42
Intense debulking (rotational atherectomy/coronary lithotripsy)	24 (14.0)	2 (4.1)	6 (14.2)	5 (12.5)	11 (26.8)	**0.005**
Intravascular lithotripsy	16 (9.3)	1 (2.0)	5 (11.9)	3 (7.5)	7 (17.0)	**0.03**
Rotational atherectomy	8 (4.6)	1 (2.0)	2 (4.7)	2 (5.0)	3 (7.3)	0.26
MACE	2 (1.5)	1 (2.0)	0	0 (0)	1 (2.4)	0.91
Creatinine levels post CTO-PCI	1.1 (0.93–1.37)	1.1 (0.97–1.64)	1.16 (0.91–1.36)	1.3 [1.1–1.6]	1.06 (0.89–1.5)	0.77
Contrast-induced nephropathy	1 (0.5)	1 (2.0)	0 (0)	0 (0)	0 (0)	0.99

**Table 4:** Univariable and multivariable logistic regression analysis for intense debulking usage.

Variable	Univariable Analysis	*p* Value	Multivariable Analysis	*p* Value
Coronary imaging	1.7 (1.2–2.7)	**0.006**	1.6 (1.02–2.4)	**0.04**
J-CTO score	2.2(1.4–3.3)	**<0.001**	2.0 (1.3–3.0)	**0.001**

## Data Availability

The data presented in this study are available from the corresponding author upon reasonable request.
